# Do Short-Term Improvements in Activities of Daily Living and Instrumental Activities of Daily Living Have Association With Return to Work in Workers With Occupational Injury? From an Occupational Injury Cohort in Taiwan

**DOI:** 10.1016/j.shaw.2024.12.003

**Published:** 2025-01-03

**Authors:** Fa-Chen Lin, Chia-Pin Lin, Hung-Yi Chuang, Tse-Wei Wu, Peng-Ju Huang, Chen-Cheng Yang, Chao-Hung Kuo

**Affiliations:** 1 Department of Occupational and Environmental Medicine, Kaohsiung Municipal Siaogang Hospital, Kaohsiung Medical University, Kaohsiung, Taiwan; 2 Department of Medical Education and Research, Kaohsiung Veterans General Hospital, Kaohsiung, Taiwan; 3 Department of Occupational and Environmental Medicine, Kaohsiung Medical University Hospital, Kaohsiung Medical University, Kaohsiung, Taiwan; 4 Department of Public Health and Environmental Medicine, College of Medicine, Kaohsiung Medical University, Kaohsiung, Taiwan; 5 Department of Medical Education, Kaohsiung Chang Gung Memorial Hospital, Kaohsiung, Taiwan; 6 Department of Orthopaedic, Kaohsiung Municipal Siaogang Hospital, Kaohsiung Medical University, Kaohsiung, Taiwan; 7 Department of Internal Medicine, Kaohsiung Municipal Siaogang Hospital, Kaohsiung Medical University, Kaohsiung, Taiwan

**Keywords:** Activities of daily living (ADL), Instrumental activities of daily living (IADL), Occupational injury, Return to work (RTW)

## Abstract

**Background:**

Rates of return to work (RTW) after an occupational injury vary considerably according to a range of factors. Limited studies have been conducted on the specific correlation between RTW and functional assessments, including activities of daily living (ADL) and instrumental activities of daily living (IADL). This prospective cohort study aims to determine if a relationship exists between ADL/IADL and RTW among injured workers in Taiwan.

**Methods:**

We recruited 162 workers who reported work-related injuries from January 2023 to May 2024. The assessment of ADL was evaluated using the Barthel Index, whereas IADL was evaluated using the Lawton Instrumental Activities of Daily Living scale. ADL/IADL were assessed immediately after the injury, at 3 and 6 months postinjury. Logistic regression models were used for the connections between ADL, IADL, and RTW while considering various confounding factors.

**Results:**

The mean ADL and IADL improved significantly at both 3 and 6 months postinjury. Logistic regression analysis indicated that IADL scores at 3 and 6 months postinjury were significantly linked to RTW. ADL scores lost significance after adjustment. Age was negatively associated with RTW, whereas sex and labor insurance status showed no significant association.

**Conclusion:**

Short-term improvements in IADL are linked to successful RTW, rather than ADL for occupationally injured workers. Evaluations of IADL should be incorporated into rehabilitation plans to predict and improve RTW. Thorough rehabilitation approaches that address various aspects of functional abilities may be crucial to support successful RTW. Further studies are required to validate these results.

## 1 Introduction

In 2021, it was reported that 46,544 workers or 4.269 per 1000 workers were involved in occupational injury in Taiwan according to the Ministry of Labor, Republic of China. The incidence rate showed a 9.7% decline compared with the previous year. Over the past 10 years, reported injuries at work have gradually decreased, showing an absolute rate reduction of 0.204% [1]. The International Labour Organization (ILO) pointed out that over 395 million workers around the world experienced a nonfatal work injury in 2019 [2]. Occupational accidents have several negative impacts influencing the injured worker. Physically, injuries may result in fractures, burn, or even amputations, limiting the capability of regular activities. Mentally, suffering from pain may reduce the quality of life and cause emotional distress, including anxiety, depression, or even posttraumatic stress disorder, and worries about getting a full recovery may become a psychological burden [3]. In terms of socioeconomic impact, the ILO estimates that work-related injuries and diseases account for approximately 4% of global gross domestic product (GDP), translating to an economic burden of around $2.8 trillion annually, and the impact of these injuries can reach up to 10% of the GDP in some developing countries [4]. It was estimated that in the United States of America solely, the cost due to occupational injury adds up to 250 billion US dollars per year, including medical costs of 67 billion US dollars (27% of the total), and indirect costs were almost 183 billion US dollars (73%) [5]. For individuals, medical expenses and other general expenses create an economic load, even exacerbated by reduced income due to less working time or switching to lower-paying jobs [6]. Significant social effects on families and communities are acknowledged. The emotional toll on family members can be profound, as the injury leads to increased caregiving responsibilities, psychological distress, and financial strain [7,8]. The sustainability of social support systems is also challenged, as work-injured individuals often require more attention and assistance in the workplace as well as personal social connections [8].

Return to work (RTW) is the term used to describe the restoration of an employee's workability. This fosters both physical recovery and mental wellness, contributing to overall well-being [9]. Reported RTW rate from worldwide studies varied from 29% to 100%, with a median of 67% [10]. In a recent study, Australia and New Zealand reported an RTW rate of 87% and 88%, respectively [11]. In Taiwan, RTW rate after 1 year of occupational injury was about 78.6% [12]. Multiple factors contribute to the success rate of RTW and have been discussed widely; yet, the results have been conflicting due to differences in groups being analyzed, selection criteria, or study protocol [13,14]. Studies have shown that longer RTW days were associated with a lower rate of successful RTW [14]. The severity of the injury was considered a key factor, as less harmful accidents usually result in a higher likelihood of successful RTW and shorter periods of sickness absence [13,15]. Senior workers had a significantly lower RTW rate than younger workers probably due to labor policies encouraging older workers to retire after injuries [10]. Economically, higher monthly salaries showed a correlation with a higher RTW rate, as they may benefit from better social support [13]. Function of social support systems, including but not limited to labor organizations, may facilitate worker's RTW by providing training courses, work accommodations, and so on [16]. Other factors may be equally important but less regarded, including individuals' functional abilities, meaning that they could carry out multiple and integrated activities on a day-to-day basis, and often measured objectively by activities of daily living (ADL) or instrumental activities of daily living (IADL) parameters [17]. Limitations on activities or participation may affect RTW, suggesting that more physical limitations or restrictions pose a negative impact on RTW [13,17]. One study primarily focusing on schizophrenic adults indicated that Assessment of Motor and Process Skills (AMPS) had a moderate correlation with the level of employment [18] but may not be directly applied to the general population.

According to available literature, minimal evidence suggests that ADL or IADL is associated with RTW. However, an individual's functioning ability contributes to their working ability and therefore should be considered an important factor for returning to work [17]. One study found that in patients with mild stroke, the rate of RTW was correlated with executive functions measured by Executive Function Performance Test, which assessed the individual's performance on four instrumental daily tasks [19]. Another study found correlations between the Functional Independence Measure sit-to-stand movement and improved the rates of RTW among patients with stroke, indicating that certain tools used to measure ADL were indeed associated with successful RTW [20]. However, current studies have not considered cases of occupational injuries; therefore, our study seeks to fill this gap. Our hypothesis is that greater improvements in functional assessment scores may lead to a higher rate of RTW among occupationally injured workers. This study aimed to determine the correlation between ADL, IADL, and RTW in occupationally injured workers, identifying potential indicators for enhancing RTW results.

## 2 Materials and methods

### 2.1 Participants and study design

This was a prospective cohort study conducted in Taiwan from January 2023 to May 2024. Participants were recruited from workers who suffered from occupational injury and hospitalization in the Kaohsiung Municipal Siaogang Hospital. The injured workers were admitted to various departments, including the orthopedic surgery department, plastic surgery department, neurosurgery department, thoracic surgery department, etc. The corresponding number of participants from each department is provided in Supplement table 1. Participants' inclusion criteria were (1) Chinese workers who lived in a city or a county of Taiwan and (2) aged ≥20 years old. Participants with mental disorders previously diagnosed by medical doctors were excluded. A total of 615 workers met the inclusion criteria and were available for further investigation. If the eligible workers agreed with the terms and conditions of the survey, they could access the self-report survey or be queried by our well-trained case managers after informed consent. Of the available respondents, 162 participants consented and completed the survey. The comparison between responders' and nonresponders’ demographics is provided in Supplementary table 1. Follow-up assessments were conducted by interviewers in person during clinic visits or via telecommunications for remote participants at three intervals: immediately after injury, at 3 months postinjury, and at 6 months postinjury. The study protocol was approved by the Institutional Review Board of Kaohsiung Medical University Hospital, Taiwan. We used the cross-sectional survey data to query the participants with occupational injury, including demographic characteristics, labor status, labor insurance, and the site of injury. Furthermore, we used the follow-up longitudinal data to query the status of RTW, ADL, as well as IADL. RTW in this study was defined as any form of returning to the workplace, whether it was resuming full duties or performing lighter, modified tasks. Fitness for RTW was certified by board-certified occupational medicine doctors, ensuring the consistency of our results.

### 2.2 ADL and IADL

ADL was assessed by the worker's dependence level through the Barthel index (BI) [21]. This tool evaluates basic self-care tasks essential for independent living. It consists of 10 tasks with a scoring system of 0–100, a higher score of BI indicates a better ability for the patient to live independently. The advantages of BI are completeness, great sensitivity, suitability for statistical manipulation, and familiarity. Other strengths include its widespread use and ease of application [22]. For IADL, we adapted the Lawton Instrumental Activities of Daily Living scale [23]. Compared with ADL, IADL focuses on more complex interactions to support daily life, which are essential for executive functions such as planning, problem-solving, or mental flexibility [24]. This scale is commonly used to assess functional decline in elderly populations and guide early interventions accordingly [23]. It consists of eight daily abilities with a maximum score of 24, and a higher score indicates better independence of the worker.

### 2.3 Statistics

Descriptive statistics, including means, standard deviations, and frequency distributions, were analyzed to summarize the demographic data. To further investigate the association between physical function and RTW status, we conducted logistic regression analyses. These analyses assessed the relationships between RTW, ADL, IADL, and potential confounding factors, such as age, gender, and labor insurance status. This approach allowed us to identify significant predictors and control for confounding variables. All statistical analyses were performed using SPSS version 21 (IBM), ensuring the robustness and reliability of our results. Statistical significance was defined as a *p*-value of <0.05 for all tests.

## 3 Results

Table 1 presents the demographic data of our study. The study cohort comprised 162 participants, primarily male accounted for 75.3% or 122 individuals, whereas females accounted for 24.7% or 40 individuals. The age distribution was as follows: 63 participants (38.9%) were aged between 20 and 39 years, 65 participants (40.1%) were aged between 40 and 59 years, and 34 participants (21.0%) were aged >60 years. The study participants came from various industry sectors: 61 (37.7%) were in manufacturing, 26 (16.0%) in construction, 26 (16.0%) in transportation and storage, 9 (5.6%) in wholesale and retail trade, 8 (4.9%) in support service activities, and 32 (19.8%) in other categories. A subgroup analysis of RTW status by industry category is provided in Supplementary table 2. In terms of the site of injury, 32 participants (19.8%) had injuries to the head and neck, 58 (35.8%) to the upper limbs, 34 (21.0%) to the lower limbs, and 38 (23.5%) to the trunk. Rehabilitation was undertaken by 31 participants (19.1%), which indicates active and regular participation in a hospital outpatient or clinic rehabilitation program. On the other hand, 98 (60.5%) had no engagement in any rehabilitation activities during the study period. The status was unclear for 33 participants (20.4%) since information on rehabilitation participation was incomplete or had no response. Labor insurance coverage was reported by 113 participants (69.8%), whereas 46 (28.4%) did not have labor insurance, and it was unclear for three participants (1.9%). Regarding RTW, 134 participants (82.7%) returned to work, 25 participants (15.7%) did not return, and three participants (1.9%) retired.

**Table 1:** Demographic characteristics of the return-to-work cohort

Characteristics (*n* = 162)	Number	%
**Sex**		
Male	122	75.3
Female	40	24.7
**Age (years)**		
20–39	63	38.9
40–59	65	40.1
≥60	34	21.0
**Industry category**		
Manufacturing	61	37.7
Construction	26	16
Transportation and storage	26	16
Wholesale and retail trade	9	5.6
Support service activities	8	4.9
Others	32	19.8
**Site of injury**		
Head and neck	32	19.8
Upper limbs	58	35.8
Lower limbs	34	21.0
Trunk	38	23.5
**Rehabilitation**		
Yes	31	19.1
No	98	60.5
Unclear	33	20.4
**Labor insurance**		
Yes	113	69.8
No	46	28.4
Unclear	3	1.9
**Return to work**		
Yes	134	82.7
No	25	15.7
Retired	3	1.9

Table 2 presents a comparative analysis of ADL and IADL between the initial evaluation immediately after injury and subsequent assessments at 3 months and 6 months postinjury within the RTW cohort. The mean ADL score significantly increased from 80.00 at the initial assessment to 96.54 at 3 months postinjury (*p* < 0.001) and 79.85–98.64 at 6 months postinjury (*p* < 0.001). Similarly, the mean IADL score improved from 20.54 initially to 23.05 at 3 months postinjury (*p* < 0.001) and 20.73–23.66 at 6 months postinjury (*p* < 0.001), indicating a significant enhancement.

**Table 2:** Changes in ADL and IADL scores in the return-to-work cohorts

Number of participants (n)			Mean	SD	*p*
*n* = 136	ADL	Immediately after injury	80.00	20.73	<0.001
		3 months postinjury	96.54	10.79	
	IADL	Immediately after injury	20.54	3.95	<0.001
		3 months postinjury	23.05	2.44	
*n* = 137	ADL	Immediately after injury	79.85	20.06	<0.001
		6 months postinjury	98.64	8.81	
	IADL	Immediately after injury	20.73	3.63	<0.001
		6 months postinjury	23.66	1.44	

ADL, activities of daily living; IADL, instrumental activities of daily living; SD, standard deviation.

Table 3 presents the logistic regression analysis of RTW, ADL, IADL, and potential confounding factors across five models. The crude model includes crude odds ratios (OR), whereas Models 1–4 present adjusted OR adjusted by various factors, including age, sex, rehabilitation, site of injury, and labor insurance. For age, the crude OR was 0.957 (95% confidence interval [CI]: 0.920–0.996), indicating a significant negative association with RTW. This association remained significant in Models 1, 2, and 4. Nevertheless, the significance was lost in Model 3. Sex showed no significant association with RTW across all models. For males, the crude OR was 1.536 (95% CI: 0.555–4.251), and adjusted ORs from Model 1 to Model 4 all lacked statistical significance. Rehabilitation status indicated a significant negative association in the crude analysis (OR 0.328, 95% CI: 0.121–0.890, *p* < 0.05) in Model 1 and in Model 4. However, this significance was not maintained in Models 2 and 3. Site of injury did not show a consistent significant association with RTW across the models. The crude ORs for head and neck, upper limbs, and lower limbs were 1.120 (95% CI: 0.308–4.067), 3.453 (95% CI: 0.814–14.639), and 1.120 (95% CI: 0.308–4.067), respectively. Adjusted ORs varied without achieving statistical significance across all models. Labor insurance status also did not exhibit significant associations with RTW in any of the models. The crude OR for those with labor insurance was 0.617 (95% CI: 0.190–1.997), with adjusted ORs in Model 1 to Model 4 lacking statistical significance.

**Table 3:** Logistic regression analysis of ADL, IADL, and potential confounders on return to work

Variables		Model 1	Model 2	Model 3	Model 4
	Crude OR (95%CI)	Adjusted OR (95% CI)	Adjusted OR (95% CI)	Adjusted OR (95% CI)	Adjusted OR (95% CI)
Age	**0.957 (0.920–0.996) ∗**	**0.945 (0.902–0.990) ∗**	**0.950 (0.907–0.996) ∗**	0.957 (0.906–1.011)	**0.868 (0.773–0.975) ∗**
Sex					
Male	1.536 (0.555–4.251)	0.995 (0.306–3.230)	0.292 (0.048–1.770)	0.217 (0.030–1.591)	0.040 (0.001–2.550)
Female (reference)					
Rehabilitation					
Yes	**0.328 (0.121–0.890) ∗**	**0.255 (0.081–0.800) ∗**	0.354 (0.100–1.250)	0.565 (0.126–2.535)	**0.059 (0.006–0.570) ∗**
No (reference)					
Site of injury					
Head and neck	1.120 (0.308–4.067)	0.755 (0.177–3.229)	0.711 (0.137–3.685)	0.474 (0.082–2.739)	0.539 (0.033–8.769)
Upper limbs	3.453 (0.814–14.639)	3.559 (0.772–16.402)	2.211 (0.450–10.866)	1.441 (0.203–10.222)	9.855 (0.617–157.483)
Lower limbs	1.120 (0.308–4.067)	1.034 (0.251–4.266)	1.333 (0.310–5.734)	2.374 (0.371–15.192)	1.211 (0.094–15.551)
Trunk (reference)					
Labor insurance					
Yes	0.617 (0.190–1.997)	0.494 (0.131–1.862)	0.390 (0.090–1.694)	0.313 (0.050–1.983)	0.657 (0.062–6.936)
No (reference)					
ADL (1)	**1.040 (1.016–1.064) ∗∗**		1.004 (0.964–1.046)		
IADL (1)	**1.205 (1.080–1.345) ∗∗**		1.243 (0.966–1.599)		
ADL (2)	**1.149 (1.063–1.243) ∗∗**			1.110 (0.999–1.232)	
IADL (2)	**1.469 (1.173–1.839) ∗∗**			**1.490 (1.029–2.158) ∗**	
ADL(3)	1.266 (1.000–1.602)				1.101 (0.880–1.376)
IADL (3)	**2.487 (1.240–4.991) ∗**				**4.189 (1.133–15.490) ∗**

(1) Immediately after injury; (2) 3 months postinjury; (3) 6 months postinjury.

Covariates adjusted for in Models 1–4: age, sex, rehabilitation, site of injury, labor insurance.

ADL, activities of daily living; CI, confidence interval; IADL, instrumental activities of daily living; OR, odds ratio.

∗ indicates a significance of *p* < 0.05; ∗∗ indicates a significance of *p* < 0.001.

ADL and IADL scores were significantly associated with RTW in various models. ADL immediately after injury had a crude OR of 1.040 (95% CI: 1.016–1.064, *p* < 0.001), whereas IADL immediately after injury had a crude OR of 1.205 (95% CI: 1.080–1.345, *p* < 0.001). The significant associations between ADL/IADL and RTW shown in crude models were seen in the assessment at 3 months postinjury and at 6 months postinjury as well, except for ADL at 6 months postinjury. In adjusted models, significant associations were observed for IADL assessed at 3 months postinjury in Model 3 (adjusted OR 1.490, 95% CI: 1.029–2.158, *p* < 0.05) and IADL evaluated at 6 months postinjury in Model 4 (adjusted OR 4.189, 95% CI: 1.133–15.490, *p* < 0.05). The association for ADL lost significance after adjustments.

## 4 Discussion

This study includes 162 individuals who suffered from occupational injury between January 2023 and May 2024 to investigate the factors associated with RTW, including the impact of age, sex, rehabilitation, site of injury, labor insurance, ADL, and IADL. Overall, the analysis indicates significant associations observed in some models between age, rehabilitation, ADL, and IADL with RTW, whereas sex, site of injury, and labor insurance do not show consistent significant associations. Although this study primarily emphasizes ADL and IADL, other factors are also analyzed thoroughly. To compare our results with currently available literature, we searched for evidence-based studies published in recent years. A best evidence synthesis by Cancelliere et al included 56 systematic reviews, half of which consisted of musculoskeletal disorders and the other half with various health conditions. They found that factors associated with positive RTW rate were higher education and socioeconomic status, higher self-efficacy and optimistic expectations for recovery and RTW, lower severity of the injury/illness, RTW coordination, and multidisciplinary interventions that include the workplace and stakeholders. In contrast, factors including older age, being female, higher pain or disability, depression, higher physical work demands, previous sick leave and unemployment, and activity limitations were categorized as negative factors for RTW. Activity limitations were defined as limited ability to perform ADL and periods of unemployment [13]. Another systematic review carried out by Etuknwa et al included 79 studies of workers with musculoskeletal disorders and common mental disorders to investigate personal and social factors influencing sustainable RTW, which was characterized by 3 months without relapse or sickness absence reoccurrence. They concluded that factors such as support from line managers or supervisors and coworkers, positive attitude, self-efficacy, young age, and higher education levels contributed to sustainable RTW [25].

Age emerged as a significant predictor of RTW in several models. Specifically, the negative association between age and RTW was evident in the crude model and Models 1, 2, and 4. This indicates that younger individuals are more likely to RTW postinjury, which aligns with existing literature, suggesting that younger age is associated with better recovery outcomes and a higher likelihood of reemployment [10,13,16,26]. Previous studies showed that workers aged >46 years were associated with lower RTW rates, and this could be explained by labor policies, which suggest older workers retire to provide vacancies for younger employees [10]. Another perspective regarding this negative association was workers will experience an extended length of disability as age increases, as more than 2.5 weeks difference in disability was observed across ages 18–80 years, hence prohibiting the success of RTW for elderly workers [26]. To sum up, our study further validates the relationship between age and RTW, demonstrating that aging is a significant negative factor influencing successful RTW. Our findings indicate that as individuals age, their probability of resuming work decreases, highlighting the importance of considering age-related factors in occupational rehabilitation programs and policies.

The study found no significant differences in RTW between males and females across all models. This suggests that sex, independent of other factors, does not significantly influence the likelihood of returning to work. The relationship between gender and the success rate of RTW was controversial, and the results were conflicting among currently available literature. An original study investigating patients with musculoskeletal disorders who participated in rehabilitation programs showed that men had a higher likelihood of RTW in the long term (10-year follow-up) [27]. Compared with our study, we included all injured patients for data analysis, regardless of participation in rehabilitation or not, thus explaining the varying results. A recent systematic review pointed out that the effect on RTW of sex was inconsistent [25]. Additional factors may heavily influence the impact of gender, such as the site of injury or the type of occupation involved, and the factors may also vary between different genders [25]. These findings emphasize the necessity for further research to pinpoint the factors influencing RTW outcomes for both men and women.

Labor insurance was not associated with RTW in any of the models. This finding suggests that labor insurance status alone does not significantly impact the likelihood of returning to work but rather is affected by other various models in our population. In Taiwan, National Health Insurance provides comprehensive coverage for all citizens; thus, the need for labor insurance is reduced. This universal health coverage ensures that individuals receive necessary medical care regardless of their employment status, diminishing the role that labor insurance plays in facilitating RTW outcomes.

There are various methods to assess an individual's work capability, ranging from specific to integrated [28]. Although specific assessment tools can provide detailed evaluations, their applicability is relatively limited. In addition, they may not be able to comprehensively assess individuals with multiple work capability impairments. On the other hand, ADL is more of an integrated method that serves as a universal assessment tool to evaluate whether patients experience limitations in their daily lives. This tool provides a comprehensive evaluation of both physical and cognitive functions. Previous studies stated that physical ability was positively correlated with workability, including the elderly population and patients who suffered from injury or medical illness [13,29]. In our study, ADL scores immediately after injury were significant in the crude model, indicating that higher functional independence in daily activities at baseline was associated with an increased likelihood of returning to work. For an individual to restore workability, they must possess the ability to accomplish daily tasks independently [17]. This significance, however, did not persist in Model 2, suggesting that the initial ADL's effect was moderated by other factors such as sex, rehabilitation, site of injury, etc. ADL scores at 3 months and 6 months postinjury showed significant associations in the crude model, but after adjusting for additional variables, the significance was not observed in Model 3 and Model 4 as well. Improvements in ADL over the short term may be positively correlated with RTW but were influenced by other confounding factors. We assume that ADL is associated with the severity of the injury or illness secondary to the occupational incident and a higher score of ADL immediately after injury translates to a less severe impact, making RTW more accessible to these workers. However, the data of our study suggest otherwise, showing the improved RTW may be a result of other variables rather than ADL.

Similar to ADL, IADL aims to assess a patient's ability to manage daily tasks. However, IADL encompasses more complex activities and is closely related to the patient's capacity to live independently within the community and thus may serve as a more accurate tool for workability. IADL scores immediately after injury were significantly correlated with RTW in the crude model, suggesting that higher initial functional independence in instrumental activities was associated with a higher likelihood of returning to work. However, this association was diminished in Model 2, which considered additional variables influencing the relationship between IADL immediately after injury and RTW. IADL scores at 3 months postinjury remained significant in the crude model and Model 3. This consistent significance highlights that improvements in IADL over the short term are crucial for RTW. Similarly, IADL scores at 6 months postinjury also showed strong significance in the crude model and Model 4. The ability to perform complex ADL, which are often critical for job-related tasks, appears to be a robust predictor of returning to work at this stage. Continued enhancements in IADL over an extended duration exhibit a significant correlation with successful RTW. One study conducted by Schwingel et al found that compared with nonvolunteering retired workers, volunteering workers and workers who still work had significantly higher IADL independence [30], which pointed out that IADL had a positive correlation with workability. Higher IADL scores represent better functional independence, which correlates with job performance and therefore is crucial for successful RTW [17]. Having a preserved functional capacity, such as meal preparation, financial management, and transportation, allows workers to engage in daily challenges and remain competitive in labor activities, thus ensuring their active participation in employment. Conversely, lower IADL scores often correlate with difficulties in performing job-related tasks. For example, workers who struggle with making phone calls may also face challenges in collaborating with colleagues. Interaction with others in the workplace encourages essential cooperative and interactive relationships, which is a crucial component across a range of occupations.

Combining the above points, we can assume that significant improvement in ADL alone is insufficient to increase the success rate of RTW for individuals. This is because ADL represents the minimal threshold for independent living. Even if the conditions of ADL are met, individuals may still lack comprehensive abilities or the capacity to utilize the tools necessary for successful reintegration into the workforce. For instance, workers in the retail industry may not only need to maintain a professional appearance, which requires adequate ADL abilities, but also proficiency in handling financial transactions. In contrast, the results regarding IADL demonstrate a correlation with RTW, a relationship that persists even at the 6-month assessment timing. This indicates that more comprehensive assessment tools such as IADL are required to ascertain whether individuals possess the necessary capabilities for returning to work. On the other hand, IADL can serve as a preliminary screening method, whereby individuals who show limited improvement in IADL are likely to have reduced chances for RTW.

The findings may assist with formulating rehabilitation programs, providing essential aid for workers to return to the workforce. Several strategies can be implemented to enhance IADL. Occupational therapy interventions may include tailored, task-specific training, which has evidence of improving IADL [[31], [32], [33]]. For instance, handwriting can be improved by strengthening the upper extremities and engagement in writing activities, subsequently enhancing skills of financial transactions [31]. Other occupational therapy interventions may be composed of multicomponent interventions, self-management, or home-based interventions [32,33]. Physical activity programs, such as resistance training and aerobic exercise, help with mobility and participation in daily chores [32]. Simulated IADL programs aimed at replicating daily functional tasks had evidence of improving IADL as well as physical fitness [34]. By implementing specific interventions for work-injured workers, we may expect successful RTW by IADL improvements. The rehabilitation professionals should have a solid understanding of IADL measurements since continuous assessment of IADL scores allows for personalized adjustments to treatment [32].

The strengths of deploying IADL for RTW assessments include its comprehensive evaluation of complex daily living skills, ease of administration, and an objective scoring scale that facilitates both comparisons between individuals and tracks changes over time for the same patient. The use of IADL has several limitations as well. The assessment is influenced by other health conditions, such as cognitive impairment or other chronic diseases [35]. Cultural differences may also affect the result, as certain tasks may be performed differently across cultures; thus, adapted versions suitable for specific populations have been proposed [35,36]. These limitations underscore the importance of careful consideration when using the IADL as a tool for RTW assessment.

The generalizability of applying IADL for occupationally injured workers' RTW evaluation can be addressed in the following aspects. First, our study's population is mainly composed of Chinese workers. Cultural differences can influence IADL relevance as well as RTW determinants, and application to other regions may require caution. Further studies in diverse populations could strengthen validity. Second, our study excluded workers with previously diagnosed mental disorders. The rationale was that IADL assessment may be substantially affected by these disorders [37], interfering with interpretation. Before applying IADL as a predictive tool, these conditions should be excluded first. Finally, well-trained evaluators must be involved for a standardized score to ensure consistency of IADL assessments. This is particularly important for accurately predicting successful RTW, as trained evaluators can minimize reporting bias and ensure the reproducibility of IADL results. This also includes using the culturally appropriate version of the IADL tool based on the evaluator's expertise.

This is the first study investigating the relationship between ADL, IADL, and RTW, which provides novel insights into how functional abilities impact the likelihood of RTW. We evaluated our participant's abilities immediately after injury and at 3 months and 6 months postinjury, providing a more in-depth analysis throughout our study. To exclude possible confounding factors, we used logistic regression models to avoid several factors impacting the interpretation.

The study has several limitations. First, the sample of 162 participants may not be representative of all workers who sustain occupational injuries. To address the relatively small sample size, our study required at least 6 months of follow-up for each case, and those who were lost to follow-up midway could not be included in the cohort. We made our best effort to recruit the participants within a 2-year period across the hospital. Nevertheless, we acknowledge that to validate these results, additional participant recruitment and a broader range of conditions are needed. Second, reporting bias may occur when evaluating ADL and IADL, which primarily relied on self-reported questionnaires. However, well-trained investigators supervised this process and took responsibility in querying the participants. ADL and IADL are scales that can be assessed objectively as well; thus, we consider this bias as minimal. Last but not least, while we took active measures to avoid possible confounding factors, such as adjustment for several variables (age, sex, rehabilitation, site of injury, and labor insurance), there may still be other unmeasured confounding factors influencing RTW outcomes. As mentioned previously, various factors may also affect RTW, including personal income, social support, work accommodation, severity of the injury, etc. Due to the complex nature of RTW determinants, not all the factors were included in our questionnaires. Additional consideration of these factors may further strengthen the findings of the study. Overall, the limitations discussed provide a valuable direction for further research.

This study recruited 162 individuals who required medical attention for work-related injuries from January 2023 to May 2024 and analyzed their demographic details, ADL, IADL, and RTW status. While ADL did not show a significant correlation with RTW, a notable positive correlation was found between IADL and RTW. Our study contributes to the field by identifying IADL improvements as a significant predictor of RTW, implying that IADL could be used as an evaluation tool in occupational rehabilitation protocols. Targeted interventions for RTW may benefit from our study, as IADL represents the fundamental abilities required by workers. Consequently, a more comprehensive rehabilitation strategy could be established to encourage favorable RTW results. Further studies may be needed to validate these results and investigate across diverse populations as well as other elements that affect RTW.

## CRediT authorship contribution statement

**Fa-Chen Lin:** Writing – review & editing, Writing – original draft, Visualization, Validation, Investigation, Formal analysis. **Chia-Pin Lin:** Software, Resources, Investigation, Formal analysis, Data curation. **Hung-Yi Chuang:** Supervision, Methodology, Investigation, Formal analysis, Conceptualization. **Tse-Wei Wu:** Writing – review & editing, Writing – original draft, Visualization, Validation. **Peng-Ju Huang:** Supervision, Resources, Methodology, Conceptualization. **Chen-Cheng Yang:** Writing – review & editing, Writing – original draft, Visualization, Validation, Resources, Methodology, Investigation, Funding acquisition, Formal analysis, Data curation, Conceptualization. **Chao-Hung Kuo:** Supervision, Resources, Methodology, Conceptualization.

## Ethical Considerations and Disclosures

This study was approved by the Institutional Review Board of Kaohsiung Medical University Hospital, Taiwan [IRB number: KMUHIRB-E(I)-20240095].

## Funding sources

This work was funded by the National Science and Technology Council, R.O.C., Taiwan (supported number: NSTC 113-2314-B-037-073), and Kaohsiung Municipal Siaogang Hospital (grant number. O-11217, R-11101), from the Occupational Injury and Disease Diagnosis and Treatment Medical Institutions Accreditation Management Subsidy from Occupational Safety and Health Administration, Ministry of Labor, R.O.C., Taiwan.

## Data availability

The original contributions presented in the study are included in the article/supplementary material, and further inquiries can be directed to the corresponding author.

## Conflicts of interest

No conflict of interest is required to be declared.
