# Supplementary material for: Do Short-Term Improvements in Activities of Daily Living and Instrumental Activities of Daily Living Have Association With Return to Work in Workers With Occupational Injury? From an Occupational Injury Cohort in Taiwan
Source: Saf Health Work. 2025 Jan 3;16(1):90–6. doi: 10.1016/j.shaw.2024.12.003 (PMC11959554; doi:10.1016/j.shaw.2024.12.003)
Supplement: Multimedia component 1 [file mmc1.docx]

**Supplementary Table 1**. Comparison of responders’ and non-responders’ demographic characteristics.

|  | **Responders** | **(N=162)** | **Non-Responders** | **(N=453)** | **Chi-square test** |
| --- | --- | --- | --- | --- | --- |
|  | **Number** | **%** | **Number** | **%** | **P-value** |
| **Sex** |  |  |  |  |  |
| Male | 122 | 75.3% | 316 | 69.8% | 0.18 |
| Female | 40 | 24.7% | 137 | 30.2% |  |
| **Age (years)** |  |  |  |  |  |
| 20-39 | 63 | 38.9% | 146 | 32.2% | 0.245 |
| 40-59 | 65 | 40.1% | 213 | 47% |  |
| Above 60 | 34 | 21.0% | 94 | 20.8% |  |
| **Industry category** |  |  |  |  |  |
| Manufacturing | 61 | 37.7% | 171 | 37.7% | 0.116 |
| Construction | 26 | 16.0% | 99 | 21.9% |  |
| Transportation and storage | 26 | 16% | 39 | 8.6% |  |
| Wholesale and retail trade | 9 | 5.6% | 29 | 6.4% |  |
| Support service activities | 8 | 4.9% | 29 | 6.4% |  |
| Others | 32 | 19.8% | 115 | 25.4% |  |
| **Site of injury** |  |  |  |  |  |
| Head and neck | 32 | 19.8% | 70 | 15.5% | 0.05 |
| Upper limbs | 58 | 35.8% | 140 | 30.9% |  |
| Lower limbs | 34 | 21.0% | 147 | 32.5% |  |
| Trunk | 38 | 23.5% | 96 | 21.2% |  |
| **Department** |  |  |  |  |  |
| Orthopedic surgery | 79 | 48.8% | 236 | 52.1% | 0.426 |
| Plastic surgery | 43 | 26.5% | 119 | 26.3% |  |
| Neurosurgery | 27 | 16.7% | 56 | 12.4% |  |
| Thoracic surgery | 9 | 5.6% | 25 | 5.5% |  |
| Surgical ICU | 2 | 1.2% | 15 | 3.3% |  |
| General surgery | 2 | 1.2% | 2 | 0.4% |  |

**Supplementary Table 2**. RTW status by industry category.

|  | **RTW** | **Non-RTW** | **Fisher’s Exact Test** |
| --- | --- | --- | --- |
| Industry category | **Number** | **Number** | **P-value** |
| Manufacturing | 50 | 9 | 0.929 |
| Construction | 22 | 9 |  |
| Transportation and storage | 21 | 9 |  |
| Wholesale and retail trade | 6 | 9 |  |
| Support Service activities | 6 | 9 |  |
| Others | 29 | 9 |  |
